# Fucosylated Antigens in Cancer: An Alliance toward Tumor Progression, Metastasis, and Resistance to Chemotherapy

**DOI:** 10.3389/fonc.2018.00039

**Published:** 2018-02-23

**Authors:** Athanasios Blanas, Neha M. Sahasrabudhe, Ernesto Rodríguez, Yvette van Kooyk, Sandra J. van Vliet

**Affiliations:** ^1^Department of Molecular Cell Biology and Immunology, Cancer Center Amsterdam, VU University Medical Center, Amsterdam, Netherlands

**Keywords:** cancer, glycosylation, fucosylation, fucosyltransferases, Lewis antigens

## Abstract

Aberrant glycosylation of tumor cells is recognized as a universal hallmark of cancer pathogenesis. Overexpression of fucosylated epitopes, such as type I (H1, Lewis^a^, Lewis^b^, and sialyl Lewis^a^) and type II (H2, Lewis^x^, Lewis^y^, and sialyl Lewis^x^) Lewis antigens, frequently occurs on the cancer cell surface and is mainly attributed to upregulated expression of pertinent fucosyltransferases (FUTs). Nevertheless, the impact of fucose-containing moieties on tumor cell biology is not fully elucidated yet. Here, we review the relevance of tumor-overexpressed FUTs and their respective synthesized Lewis determinants in critical aspects associated with cancer progression, such as increased cell survival and proliferation, tissue invasion and metastasis, epithelial to mesenchymal transition, epithelial and immune cell interaction, angiogenesis, multidrug resistance, and cancer stemness. Furthermore, we discuss the potential use of enhanced levels of fucosylation as glycan biomarkers for early prognosis, diagnosis, and disease monitoring in cancer patients.

## Introduction

The abnormal cell growth and the potential to invade or spread to other tissues of the body is what characterizes cancer. Aberrant glycosylation has been recently proposed as universal aspect of this disease. Despite the notable absence of this critical post-translation modification of proteins and lipids from both the original (1) and the next-generation (2) hallmarks of neoplastic malignancies, altered glycosylation is causally associated with the acquisition of all characteristic features of tumor cells (3).

Hakomori and Kannagi were the first to describe the incomplete and neo-synthesis processes defining tumor-specific glycosylation (4). Cancer cells often display differential expression levels of critical glycans or distinct carbohydrate epitopes that are not present in their normal counterparts. Increased fucosylation, truncated *O*-glycans, and increased sialylation are a well-established signature of malignant cell transformation (5). Importantly, these alterations greatly affect tumor cell–cell adhesion, cell–matrix interactions, cell-signaling, metabolism, angiogenesis, and immune modulation, eventually leading to cancer progression and metastasis.

Fucosylation represents the transfer of a fucose residue (from GDP-fucose) to oligosaccharide chains carried by cell-surface glycoproteins or glycolipids (6). It is regulated by a number of molecules, such as GDP-fucose synthetic enzymes, GDP-fucose transporters, and fucosyltransferases (FUTs). Two types of fucosylation exist, depending on the site of the oligosaccharide chain to which the fucose is added: core fucosylation and terminal fucosylation. Today, the biological role of enhanced fucosylation during inflammation and cancer is gaining more attention (7), since changes in fucosylation can facilitate the development of novel strategies for early prognosis, diagnosis, and therapy (8).

**Figure 1:**
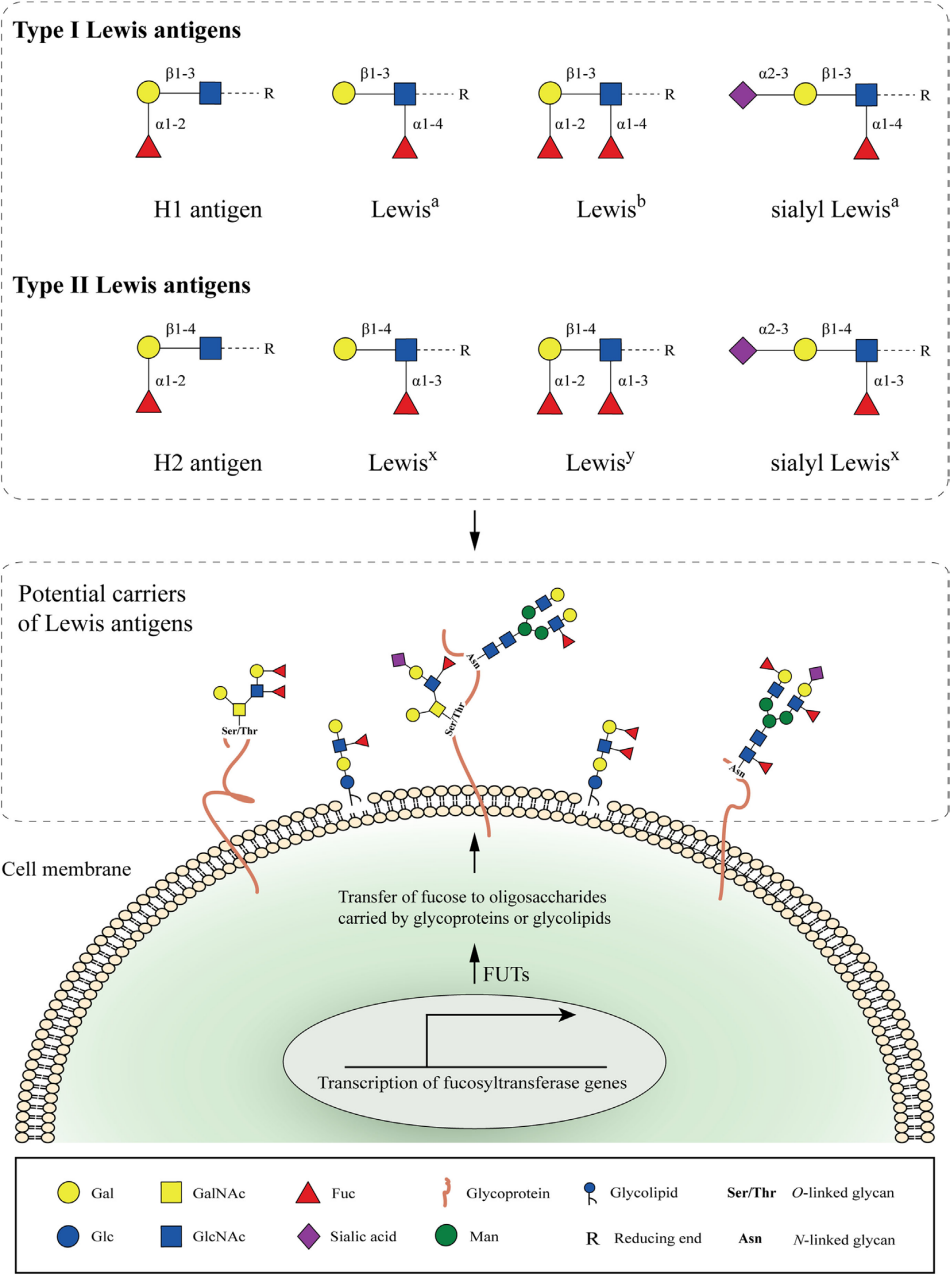
Cell-surface fucosylated antigens. Type I (H1, Lewis^a^, Lewis^b^, and sialyl Lewis^a^) and type II (H2, Lewis^x^, Lewis^y^, and sialyl Lewis^x^) Lewis antigens are terminal fucosylated carbohydrate motifs decorating cell surface glycoproteins or glycolipids. In the case of glycoproteins, *N*- and *O*-linked glycans containing Lewis antigens are covalently attached to the protein at Asparagine (Asn), or Serine and Threonine (Ser/Thr) residues, respectively. Expression of Lewis antigens is attributed to the expression of key enzymes, named fucosyltransferases (FUTs). The substrate specificity of different FUTs determines the site-specific transfer of fucose to oligosaccharides and the synthesis of the respective Lewis determinants.

Type I and type II Lewis antigens are terminal fucosylated carbohydrate epitopes belonging to the human histo-blood group antigen system, which is generally known as the Lewis antigen system (9). H1, H2, Lewis^a^ (Le^a^), Lewis^b^ (Le^b^), Lewis^x^ (Le^x^), and Lewis^y^ (Le^y^) are all structurally related members of this system (Figure 1). The same three monosaccharide units are present in all Lewis determinants, namely, *N*-acetylglucosamine (GlcNAc), galactose (Gal), and fucose (Fuc), which differ only in their corresponding glycosidic bonds (Galβ1-3GlcNAc in type I and Galβ1-4GlcNAc in type II Lewis antigens). Further addition of sialic acids to these epitopes can give rise to more complex glycan structures, such as sialyl Lewis^a^ (sLe^a^ or CA19-9) (10) and sialyl Lewis^x^ (sLe^x^) (11) (Figure 1).

According to the species-, tissue-, or cell-specific expression, early studies have shown that Lewis antigens are involved in various intercellular and intracellular biological processes, including cell adhesion and cell communication events during embryogenesis and later development (9). However, pronounced overexpression of Lewis epitopes has been reported in many types of cancers (12). Specifically, high-density expression of fucosylated antigens by carcinomas, such as colorectal cancer, is attributed to the increased expression of relevant FUTs and is correlated with poor prognosis and decreased survival (13).

We here review the impact of Lewis antigen overexpression by tumor cells on multiple biological aspects related to cancer development and progression. Great emphasis is given to the potential role of fucosylated epitopes as cancer biomarkers and to their involvement in the increased proliferative, invasive, and metastatic capacity of cancer cells. Also, we discuss in detail the implications of Lewis antigens in endothelial to mesenchymal transition (EMT), in the interaction of cancer cells with endothelial and/or immune cells and in the induction of multidrug resistance and cancer stemness. Throughout this review, we will highlight the significance of a fine-tuned expression of Lewis moieties, since their uncontrolled appearance on the cancer cell surface can have detrimental effects on tumor growth and the subsequent communication with their surrounding tissue microenvironment.

## Specificity of FUTs

So far, 13 FUT genes have been identified and characterized in humans. The enzymes that are coded by these genes can be categorized into five groups, according to the type of linkage of the added fucose residue. FUT1 (H enzyme) and FUT2 (Se enzyme) are α1-2 fucosyltransferases (14), whereas FUTs3–7 together with FUT9 are α1-3 fucosyltransferases (15). Recent studies suggest that FUT10 and FUT11 belong to the α1-3 fucosyltransferase family, as well (16). Remarkably, only the FUT3 enzyme exhibits a combined α1-3 and α1-4 fucosyltransferase activity. FUT8 is responsible for the production of core fucosylation (α1-6 fucosyltransferase) (17, 18), whereas Pofut1 and Pofut2 (protein *O*-fucosyltransferase 1 and 2, respectively) are enzymes specific only for *O*-fucosylation (19, 20).

The preferred sites for fucosylation differ substantially between FUTs (21), something that greatly affects the synthesis of terminally fucosylated epitopes. All the known Lewis antigen-synthesizing fucosyltransferases (FUTs1–7 and FUT9) possess a unique substrate specificity, thereby increasing the complexity and the bioavailability of fucose-containing Lewis epitopes in naturally occurring glycoconjugates. Nevertheless, the biological consequences of this complexity in different stages of human carcinogenesis are not fully elucidated yet (Table 1).

**Table 1:** Overview of Lewis antigen-synthesizing fucosyltransferases (FUTs) and their known implications in cancer.

Enzyme	Enzyme activity	Synthesized antigen	Cancer-related features
FUT1	α1-2 Fucosyltransferase	H antigen, Lewis^b/y^	Cell proliferation (80–85), endothelial to mesenchymal transition (EMT)/tissue invasion (96), metastasis (47), angiogenesis (113, 114), and resistance to chemotherapy (139)
FUT2	α1-2 Fucosyltransferase	H antigen, Lewis^b/y^	Still under investigation
FUT3	α1-3/4 Fucosyltransferase	Lewis^a/b/x/y^Sialyl Lewis^a/x^	Cell proliferation (90), EMT/tissue invasion (94, 95), metastasis (90), and cancer stemness (150)
FUT4	α1-3 Fucosyltransferase	Lewis^x^Sialyl Lewis^x^	Cell proliferation (86, 87), EMT/tissue invasion (97–99), resistance to chemotherapy (138, 140, 141), and potential cancer biomarker (78)
FUT5	α1-3 Fucosyltransferase	Sialyl Lewis^x^	Cell proliferation (88)
FUT6	α1-3 Fucosyltransferase	Sialyl Lewis^x^	Cell proliferation (88), EMT/tissue invasion (95), resistance to chemotherapy (138), and cancer stemness (150)
FUT7	α1-3 Fucosyltransferase	Sialyl Lewis^x^	Cell proliferation (88)
FUT8	α1-6 Fucosyltransferase	Core fucosylation	Tissue invasion (17) and metastasis (18)
FUT9	α1-3 Fucosyltransferase	Lewis^x^	Still under investigation

*FUTs1–7 and FUT9 display differential substrate specificity regarding the synthesis of terminally fucosylated antigens and are involved in various aspects of cancer progression. In contrast, FUT8 is responsible for core fucosylation only; however, its expression is also associated with tumor-promoting characteristics*.

## Physiological Expression of Lewis Antigens

Understanding the pattern of Lewis antigen expression in normal tissues, together with the physiological functions that these carbohydrates exert, will set the framework to understand their altered regulation and their tumor-promoting capabilities in the context of cancer.

During human embryogenesis, the appearance of fucosylated epitopes is attributed to the overexpression of certain FUT genes, such as the *Fut4* and the *Fut9* genes (22). The Lewis^x^/SSEA-1 (Stage-specific embryonic antigen-1) trisaccharide is the most representative example. Its expression begins gradually during cell differentiation in the nephric duct, nephric tubule, yolk sac, and on the surface of embryonic ectodermal cells of the epidermis, where it is known to play a vital role in cell–cell recognition and adhesion processes (23).

Lewis antigens that are moderately expressed in healthy adult tissues, such as in the mucosal epithelium of the digestive system, in the brain and by certain immune cell subsets, have similar functions, however, in a different context (24). In epithelial tissues, Lewis^x^ expression is mainly found in the stomach, colon, salivary glands, kidneys, bladder, epididymis, uterus, cervix, and medulla, while Lewis^y^ expression has been detected in epithelial cells from the breast, lung, prostate, colon, stomach, pancreas, uterus, ovary, salivary glands, and the Panneth cells of the small intestine. In contrast, sialyl Lewis^a^ is mostly expressed on normal fibroblasts, on the luminal side of ductal epithelial cells, and on some parenchymatous cells (25).

Lewis^x^ is the predominant fucosylated antigen in the brain and it facilitates cell–cell interactions involved in neuronal development, with FUT9 being the responsible Lewis^x^-synthesizing enzyme in the nervous system (26). Mice lacking the *Fut9* gene, thus fully devoid of Lewis^x^ expression in the brain, exhibit no obvious pathological differences compared to wild-type mice, but have an increase in anxiety-like behaviors (27). Currently, Lewis^x^ is still used as a surface biomarker for the identification of neural stem cells (28).

Moreover, immune cells display different fucosylated epitopes on their cell-surface. For example, expression of Lewis^x^ on human mature granulocytes (neutrophils, eosinophils, and mast cells) is attributed to FUT9 activity, whereas Lewis^x^ expression on promyelocytes is determined by FUT4 (29). In terms of function, Lewis^x^ is necessary for neutrophil transepithelial migration (30), and it exerts positive immunomodulatory effects on dendritic cells (DCs) *via* engagement of the C-type lectin receptor (CLR) dendritic cell-specific ICAM-3 grabbing non-integrin (DC-SIGN) (31, 32). Sialyl Lewis^x^ is commonly found on the surface of neutrophils and monocytes, facilitating extravasation of these cells to sites of inflammation through the interaction with E-selectin expressed by endothelial cells (11). Finally, granulocytes are the only peripheral blood immune cells that weakly express the Lewis^y^ antigen (33).

## Lewis Antigen Expression in Cancer

Overexpression of Lewis antigens, along with the respective FUT proteins, has been reported in many different types of cancers (24). Here, we summarize evidence of increased fucosylation compared to healthy tissues, as well as the known association of terminal fucosylated epitopes with each type of cancer and the tumor microenvironment (for overview see Table 2).

**Table 2:** Clinical relevance of Lewis antigen overexpression in different types of cancers.

Lewis antigen	Major protein carriers	Cancer types	Clinical relevance to cancer
Lewis^x^	Carcinoembryonic antigen (CEA) and carcinoembryonic antigen cell adhesion molecule (CEACAM) family (76), CD98 (59), and ICAM-1 (59)	Epithelial cancers, brain cancers, leukemias, and lymphomas	Decreased survival (36, 38), metastasis (39, 44, 45, 51), correlation with cancer stage (51), and cancer biomarker (58)
Lewis^y^	CEA and CEACAM family (76), carbohydrate antigen 125 (70, 71), and CD44 (153)	Epithelial cancers	Decreased survival (40), metastasis (47, 54), correlation with cancer stage (42), and cancer cell differentiation status (37, 46, 54)
Sialyl Lewis^a^	Transforming growth factor-β (TGF-β) (95), MUC1 (107), MUC5AC (107), Apo-B-100 (107), Apo-E (107), and kininogen (107)	Epithelial cancers	Decreased survival (72, 73), metastasis (72, 107) and cancer biomarker (72, 73)
Sialyl Lewis^x^	TGF-β (95), cancer antigen 15.3 (78), α-1 acid glycoprotein (107), and ceruloplasmin (107)	Epithelial cancers and leukemias	Decreased survival (36) and metastasis (45, 107)

### Lung Cancer

According to the cancer statistics of 2016, lung cancer is the most prevalent malignant disease in both sexes worldwide, ranking first in cancer mortality (34). Lung cancer can be divided into two types: small cell lung cancer and non-small cell lung cancer. The latter one accounts for almost 85% of all the cases and includes adenocarcinoma, squamous cell carcinoma, and large cell carcinoma (35). Lewis^x^ expression in lung cancer is higher in adenocarcinomas and squamous cell carcinomas, compared to small cell carcinomas. Also, overexpression of both Lewis^x^ and sialyl Lewis^x^ antigens is associated with a shortened survival time of patients (36). Besides Lewis^x^ and its sialylated isoform, the Lewis^y^ antigen is also overexpressed in non-small cell lung cancer patients and is considered as a valuable marker of cancer cell differentiation (37).

### Breast Cancer

Overexpression of fucosylated epitopes in breast cancer patients has an important prognostic value. Lewis^x^ expression is an independent prognostic factor for survival in young (<50 years) patients with triple-negative breast cancer (stages I, II, and III) and is correlated with poor recurrence-free and overall survival (38). A close association between expression of Lewis^x^ and the leading edge of the invading tumor has been reported, pinpointing to a possible role of this antigen in breast cancer metastasis (39). Furthermore, overexpression of the Lewis^y^ antigen in lymph node-negative breast cancer patients is associated with poor prognosis and a substantial decrease in survival (40).

### Colorectal Cancer

In colorectal cancer, Lewis^x^ expression on the surface of both infiltrating inflammatory cells and cancer cells increases during disease progression. Specifically, Lewis^x+^-infiltrating immune cells (predominantly neutrophils) are identified in the invasive front of the tumor mass, whereas Lewis^x+^ tumor cells are mostly located in the center (41). Lewis^y^ is highly expressed in gastrointestinal carcinomas, including colorectal cancer. It is detected in 40–50% of total cases with either gastric or colorectal malignancy, and its overexpression is correlated with increased tumor staging, especially stage IV (42).

### Hepatocellular Carcinoma (HCC)

Hepatocellular carcinoma is the primary malignant disease of the liver, whereby intrahepatic metastasis is a poor prognostic indicator of HCC (43). There is a correlation between Lewis^x^ expression and histologic intrahepatic metastasis, although the difference between Lewis^x+^ and Lewis^x−^ HCC samples in terms of patient survival are not statistically significant (44). The involvement of α1-3 fucosyltransferases and type II Lewis antigens, such as Lewis^x^ and sialyl Lewis^x^, in human liver cancer progression and metastasis has been confirmed by other studies (45). Also, a close correlation between Lewis^y^ expression in HCC cells and the degree of de-differentiation as well as the increased proliferative and metastatic potential of the whole tumor has been described (46, 47).

### Other Types of Cancers

Lewis^x^ and sialyl Lewis^x^ are also highly expressed in renal and bladder carcinomas (48). Lewis^x^ overexpression has been detected in more than 60% of the human renal cancer specimens tested and is proposed as a potential therapeutic target for renal cancer metastasis (49). In the bladder, Lewis^x^ is considered as a marker of malignant transformation (50), and its expression has been correlated with the stage, grade, and metastatic potential of the transitional cell carcinoma of the bladder (51).

The presence of both Lewis^x^ and Lewis^y^ has been reported in pancreatic cancer, too (52). Although Lewis^x^ is not expressed in normal pancreas (except for some cases of chronic pancreatitis), Lewis^x^ overexpression has been identified in 50–70% of pancreatic cancer tissues (53). In prostate cancer, high Lewis^y^ expression has been detected in localized and metastatic adenocarcinomas (54). Overall, high Lewis^y^ expression is correlated with the poor differentiation status and the metastatic potential of tumor lesions in the prostate.

Strikingly, overexpression of Lewis determinants is not merely restricted to solid tumors (55). Ball et al. used a flow cytometric analysis to assess the Lewis^x^ expression in normal and acute myeloid leukemia (AML) cells (56). In this study, AML cells displayed the highest binding with the anti-Lewis^x^ antibody PM-81. In some cases, enhanced binding was detected after neuraminidase treatment, implying that sialyl Lewis^x^ is also expressed in AML. In addition, expression of Lewis^x^ has been associated with a high risk of relapse in children with acute lymphoblastic leukemia (57). Finally, the Lewis^x^ determinant is expressed on Hodgkin’s Reed–Sternberg cells (often carried by CD98 and ICAM-1) and is an established diagnostic marker for patients with Hodgkin’s lymphoma (58, 59).

## Lewis Antigens as Cancer Biomarkers

Given that fucosylated epitopes are overexpressed in several types of cancers, a general interest in the development and further use of glycan-based tumor biomarkers exists (60). The term cancer biomarker refers to any biological molecule that is present in the blood, other body fluids, or tissues that discloses signs of malignant cell transformation. The discovery and validation of cancer-specific biomarkers are of utmost importance, since they can be exploited for the early prognosis and diagnosis of cancer patients. Also, they can be used for monitoring the tumor grade, the disease stage, and the response to treatment or estimate the risk of disease recurrence. Nonetheless, the gap between the initial development of emerging cancer biomarkers and their subsequent clinical implementation is still a big challenge in terms of sensitivity and specificity (61).

So far, a number of gene- or protein-based cancer biomarkers have been applied to the clinical management of cancer patients with BRCA1/BRCA2 (breast/ovarian cancer) (62), HER-2 (breast cancer) (63), PSA (prostate cancer) (64), and S100 (melanoma) (65) as a few representative examples. Interestingly, other established cancer biomarkers, such as carbohydrate antigen 125 (CA-125) (66), carbohydrate antigen 19.9 (CA19.9 or sialyl Lewis^a^) (67), and carcinoembryonic antigen (CEA) (68), are all strongly associated with an aberrant glycosylation profile during cancer progression and more specifically, with increased levels of fucosylation.

Carbohydrate antigen 125 or mucin 16 (MUC16) is a glycoprotein that is overexpressed in different types of cancers, although it is mainly used as a biomarker for the early prognosis of ovarian cancer (69). The detection of increased CA-125 serum levels combined with increased expression of fucosylated epitopes on it, such as Lewis^y^, is associated with worse prognosis and survival (70, 71). Moreover, CA19.9 or sialyl Lewis^a^ is the most validated serum biomarker used for the management of pancreatic cancer patients to date (72). Of note, it has been shown that CA19.9 has the highest predictive value as a stand-alone marker, although in combination with other biomarkers (such as CA-125 and CEA) it is better able to predict the outcome of patients after surgery and chemotherapy (73).

Carcinoembryonic antigen and the carcinoembryonic antigen cell adhesion molecule (CEACAM) family represent a group of glycoproteins found in high levels in the serum of patients with a wide range of tumors. They are currently used as biomarkers for the early detection among other colorectal, pancreatic, and lung cancers (74). A continuous increase in serum CEA is typically correlated with disease progression (75). Also, analytical glycoprofiling of circulating cancer-associated CEA has revealed significantly increased expression of type I and type II Lewis antigens compared to CEA from healthy individuals (76). This may possibly explain the current focus on the glycosylation status of various cancer-related protein biomarkers and on the implementation of more fucosylated epitopes (e.g., type II Lewis antigens) in the field of cancer biomarker research. For instance, increased antennarity/branching (glycan structures that are not linear and have two or more branches) combined with higher levels of the Lewis^x^ motif have been detected in serum *N*-glycoproteins derived from epithelial ovarian cancer patients (77). More specifically, tetra-antennary *N*-linked glycans bearing three Lewis^x^ moieties and tri-antennary *N*-linked glycans bearing one Lewis^x^ moiety can be found in the serum of patients, but not in the control sera, suggesting a possible role of these epitopes as useful biomarkers for ovarian cancer.

In addition, expression of FUT4, whose synthetic epitopes are Lewis^x^, Lewis^y^, and sialyl Lewis^x^, is higher in breast cancer tissues and serums compared to normal tissues and control serums, respectively. Since induction of FUT4 expression is also correlated with expression of another cancer-associated marker, the cancer antigen 15.3 (CA15.3), FUT4 has been proposed as a novel biomarker for the early prognosis of breast cancer (78). However, it is still unknown whether any of these Lewis antigens (or FUTs) can be further exploited as valuable cancer biomarkers, independent of their respective carrier molecules.

## Cell Survival and Proliferation

In general, each type of cancer is characterized by its own complexity and idiopathy. Increased expression of Lewis antigens (due to upregulation of related FUT proteins) affects several functional aspects of cancer cell biology, including EMT, the interaction with immune and endothelial cells, and the induction of multidrug resistance and cancer stemness. Nevertheless, there are certain characteristics that are shared by every single neoplastic disease, such as deregulation of the normal cell growth and proliferation *via* the induction of pro-survival and/or anti-apoptotic signaling pathways (79).

Overexpression of different FUTs and their synthesized fucosylated antigens during malignant cell transformation are correlated with the acquisition of an increased proliferative capacity and a pro-survival phenotype. For instance, transfection of the ovarian cancer cell line RMG-1 with a cDNA encoding the human *Fut1* gene results in a high cell surface expression of the di-fucosylated Lewis^y^ epitope and in a more aggressive phenotype (80). Specifically, RMG-1-hFUT1^+^ cells exhibited increased proliferation and cell cycle regulation compared to the RMG-1 wild-type cells, due to activation of the PI3K/Akt (81), ERK/MAPK (82), EGFR (83), and transforming growth factor-β1 (TGF-β1) (84) signaling pathways and stimulation of IGF-R1 expression (85).

Similarly, induction of FUT4 expression in the breast cancer cell line A431 leads to increased cell cycle progression and skews the balance toward the S-phase of the cell division process. The underlying mechanism includes activation and cross talk of the PI3K/Akt and MAPK signaling pathways (86). Overexpression of FUT4 in the breast cancer cell lines MCF-7 and MDA-MB-231 is regulated by certain transcription factors (heat-shock factor 1 and Sp1) and micro-RNAs (miR-224-3p and miR-493-5p), all of which have a direct effect on breast cancer cell proliferation and invasion again through the PI3K/Akt and ERK/MAPK pathways (87).

Besides FUT1 and FUT4, overexpression of other FUTs is also associated with the induction of the abovementioned signaling cascades. In colorectal cancer, miR-125a-3p influences cell proliferation mediated by the PI3K/Akt pathway by regulating the expression levels of FUT5 and FUT6 (88). Moreover, upregulation of FUT7 is associated with increased proliferation of the lung cancer A549 cell line through activation of the EGFR/AKT/mTOR pathway (89). Finally, knockdown of the *Fut3* gene in the prostate cancer cell line MDA PCa2b results in decreased cell growth *in vitro*; however, the exact signaling pathways involved have not been determined yet (90).

Clearly, cancer-related overexpression of different FUTs results in the induction of common pro-survival signals. Therefore, potent drugs/inhibitors of these specific signaling cascades need to be considered for future development of effective treatment of cancer patients.

## Epithelial to Mesenchymal Transition

Another important feature that cancer cells have in common is the increased tissue invasiveness and metastatic potential due to genetic or epigenetic alterations (91). EMT is a type of cellular transdifferentiation that is strongly associated with both invasion and metastasis, and it is currently an active field of cancer research (92). During this complex biological process, cancer cells of epithelial origin lose their polarity and cell–cell adhesion/interactions. They become more motile and are eventually capable of invading neighboring healthy tissues before spreading to other parts of the body. A number of different molecules regulate this transition state, such as transcription factors, cytoskeletal or cell-surface proteins, extracellular matrix enzymes, and micro-RNAs (93). Briefly, cancer-induced EMT is characterized by the downregulation of the epithelial cell marker E-cadherin together with concomitant upregulation of mesenchymal protein markers, such as N-cadherin, vimentin, and fibronectin. In parallel, transcription factors such as Snail 1/2, Twist, ZEB1/2, and matrix metalloproteinases such as MMP-2 and MMP-9 are all overexpressed in malignant cells undergoing EMT. In most cases, TGF-β signaling is involved in the establishment of the aforementioned EMT molecular signature; however, the convergence of other cancer-associated signaling cascades (Wnt, Notch, EGF, PI3k/Akt, and MEK/ERK) has proven to be essential.

Remarkably, the expression levels of different FUTs are strongly associated with the establishment of a robust EMT phenotype. First of all, treatment of the HT-29 and DLD-1 colon cancer cells with the EMT-inducing factors EGF or b-FGF results in transcriptional downregulation of FUT2, however, pronounced upregulation of FUT3 and increased cell surface expression of the sialyl Lewis^a/x^ epitopes *in vitro* (94). In this case, enhanced binding of E-selectin to colon cancer cells undergoing EMT could be observed, hinting to an increased metastatic potential of the cells.

Similar to EGF and b-FGF, TGF-β-induced upregulation of FUT3 and FUT6 also leads to overexpression of sialylated Lewis antigens in colorectal cancer cell lines and subsequently to an increased migratory phenotype (95). The ovarian cancer cell line RMG1, which overexpresses FUT1, exhibits elevated protein levels of the integrin adhesion receptor α5β1, upregulation of the matrix metalloproteases MMP-2 and MMP-9 and a concomitant downregulation of the tissue inhibitors of metalloproteinases TIMP-1 and TIMP-2 (96). Together, these findings may partially explain the invasive properties that have been observed in this cell line.

Furthermore, overexpression of FUT4 in the human non-small cell lung cancer cell lines A549, H1299, and H358 is associated with increased tissue invasiveness, metastasis, and induction of EMT (97). Treatment of these cells with the ginsenoside compound Rg3 or with short-hairpin RNA targeting FUT4 (shFUT4) reverts the mesenchymal phenotype through increased levels of E-cadherin and decreased levels of N-cadherin, vimentin, and Snail. Importantly, administration of Rg3 or treatment with shFUT4 of non-small cell lung cancer cells leads to significant inhibition of the migratory and invasive properties of the cells *in vitro*. Besides that, Rg3 administration results in a remarkable inhibition of EMT characteristics and in a decreased growth and metastatic potential of the non-small lung cancer cell tumors in a xenograft setting. Similar effects of Rg3 have also been described in melanoma, where Rg3 affects FUT4 expression and inhibits the FUT4-associated EGFR/MAPK signaling pathway (98, 99).

Finally, elevated expression levels of FUT4 in breast cancer cells are associated with the acquisition of a mesenchymal phenotype and a greater cell motility (100). More specifically, FUT4-mediated activation of the PI3K/Akt and NF-κB pathways resulted in EMT, defined in this case by the induction of Snail and MMP-9 expression. Taken together, a common, cancer-specific axis of fucosylation appears to exist, where overexpression of FUT genes is associated with the induction of pro-survival signals and increased tissue invasiveness, represented mainly by higher cell proliferation rates (as mentioned in the previous section) and by the establishment of a generalized EMT phenotype.

## Interaction with Endothelial Cells and Tumor Angiogenesis

The development of new blood vessels from a pre-existing vascular network is called angiogenesis and has a pivotal role during cancer progression (101). Tumor cells generally require enhanced blood supply in order to maintain the necessary oxygen and nutrient levels for their rapid growth. Intratumoral hypoxia is a well-established driver of tumor angiogenesis (102). Moreover, immune cells (macrophages, neutrophils, mast cells, eosinophils, T cells, B cells, and NK cells) along with stromal cells (pericytes, adipocytes, and fibroblasts) reside within the tumor microenvironment and exert potential pro-angiogenic effects through their bioactive products such as cytokines, growth factors, and secreted microvesicles (103).

During angiogenesis increased growth, migration, and differentiation of endothelial cells lining the inner wall of the newly formed blood vessels can be observed. Today, a large number of anti-angiogenic cancer therapies focus on targeting these endothelial cells (104). Interestingly, endothelial cells express Lewis antigen-binding proteins such as E-selectin (CD62E), P-selectin (CD62P), and the scavenger receptor C-type lectin (SRCL), supporting the idea that during cancer angiogenesis potential interactions between these receptors and their fucosylated ligands expressed by tumor cells exist (105).

Selectins are cell adhesion molecules that recognize and bind carbohydrate structures in a Ca^2+^-dependent manner. E-Selectin is constitutively expressed by venous endothelial cells in the bone marrow and the skin, whereas in other organs it can only be expressed upon stimulation with LPS or pro-inflammatory cytokines such as TNF-α and IL1-β. P-Selectin is expressed by both platelets and activated endothelial cells, where it is stored in the α-granules and within the Weibel–Palade bodies, respectively (106). The cancer-related epitopes sialyl Lewis^a^ and sialyl Lewis^x^ are major selectin ligands (carried by different glycoproteins, Table 2). Tumor cells expressing these antigens interact with endothelial cells expressing E/P-selectin, a process that eventually leads to cancer cell extravasation, which is crucial during metastasis (107). Besides the aforementioned determinants, Lewis^x^ expressed by malignant cells can also interact with E-selectin. For example, Lewis^x^ expressed by non-small cell lung cancer cells interacts with TNFα-induced CD62E on brain endothelial cells, facilitating the adhesion between these two cell types and promoting CNS metastasis similar to sialyl-Lewis^x^ (108, 109).

Lewis^x^ can also be recognized by endothelial cells through the SRCL (110). This receptor not only shares structural and functional similarities with type A scavenger receptors (whose main function is the removal of oxidized lipoproteins) but also has a Ca^2+^-dependent carbohydrate recognition domain. It binds the Lewis^x^ trisaccharide with high affinity, such as DC-SIGN; however, SRCL recognizes the galactose residue of Lewis^x^ instead of the fucose residue that is bound by DC-SIGN (111). This receptor is responsible for the endocytosis and degradation of glycoprotein ligands, and it is the linking component between the interaction of Lewis^x^-bearing cells and endothelial cells both in humans and mice (112). Unfortunately, the role of SRCL in cancer is not fully explored. Yet, the specificity for Lewis^x^ together with the distribution of this receptor on the vascular endothelium might indicate its involvement during cancer-induced angiogenesis or metastasis.

Finally, Lewis^y^ expressed by endothelial cells contributes to tumor-associated vascularization. Induction of endothelial FUT1 expression and increased levels of endothelial cell-surface expression of Lewis^y^ have been observed in the capillaries of tumor-infiltrated tissues (113). Also, the use of a designed Lewis^y^-saccharide mimetic has been shown to interfere with normal endothelial function and to inhibit angiogenesis *in vitro* (114). Whether Lewis^y^ overexpression by tumor cells has a direct effect on the vasculature during metastasis is not known yet. Therefore, the potential interaction of Lewis^y+^ cancer cells with endothelial cells, as well as the underlying mechanisms of such interaction, requires further investigation. Besides Lewis^y^, the implications of tumor-associated Lewis^a^ and Lewis^b^ antigens during cancer angiogenesis are still unknown.

## Interaction with the Immune System

The immune system plays a fundamental role during cancer progression. Innate and adaptive immune cells reside within the tumor microenvironment and determine the rate of the tumor growth (115). According to the immunoediting hypothesis, the relationship between the host organism and the developing tumor consists of three distinct phases: elimination of tumor cells by the immune system, an equilibrium phase between the tumor and the immune system, and tumor escape from immune destruction (116).

Although full activation of the adaptive immune system seems to be mandatory for successful tumor eradication, paradoxically, chronic activation of different types of innate immune cells within or close to the tumor site lead, inevitably, to cancer progression (117). Therefore, greater emphasis should be given to the cancer-innate immune cell axis in the context of future cancer immunotherapy (118). This will probably assist in the identification of more effective ways to manipulate and subsequently activate the adaptive immune system, thereby greatly increasing the possibility of tumor destruction.

Dendritic cells are key regulators of the anti-cancer immune response (119). DCs are capable of recognizing tumor-specific antigens and activating effector T cells, which in turn proceed to eradicate malignant cells. Altered tumor glycosylation can be sensed by DCs *via* certain glycan-binding proteins, such as the CLRs (120). Interestingly, CLRs expressed by DCs can capture tumor antigens and modulate the induction of anti-tumor immune responses through the regulation of T cell polarization (121). In fact, the molecular build-up of the antigen proves to be of utmost importance in determining the subsequent skewing toward immunity or tolerance.

Dendritic cell-specific ICAM-3 grabbing non-integrin (CD209) is a multifunctional Ca^2+^-dependent lectin receptor expressed in humans on the surface of antigen-presenting cells, such as immature DCs and macrophages (122). It specifically recognizes all the non-sialylated Lewis antigens (Lewis^a^, Lewis^b^, Lewis^x^, and Lewis^y^), and its main functions are related to cell adhesion, cell migration, antigen uptake, and subsequent antigen presentation.

In colorectal cancer, DC-SIGN on DCs recognizes the Lewis^x^ and Lewis^y^ epitopes carried by either the tumor-associated CEA or the CEACAM1 (123). Remarkably, DC-SIGN specifically binds only to CEA isolated from primary colorectal cancer cells and colon cancer cell lines and not to CEA originating from the normal colon epithelium. This can be explained by the enhanced expression of these glycan structures in malignant cells of the colon. Likewise, Lewis^a^ and Lewis^b^ epitopes displayed on CEA and CEACAM1 from colorectal cancer cells can also be recognized by DC-SIGN (124). Coculture of the colon cancer cell line SW1116 bearing Lewis^a/b^ with LPS-stimulated monocyte-derived DCs resulted in a significant increase in the well-known immunoregulatory cytokines IL-6 and IL-10, implying the acquisition of an immunosuppressive phenotype.

The exact contribution of DC-SIGN in the induction of an immunosuppressive tumor microenvironment is not fully understood yet. However, there is evidence of carbohydrate-specific signaling through DC-SIGN on DCs leading to tolerance against invading extracellular pathogens expressing Lewis epitopes (125). Specifically, DCs that interact with the fucose-expressing parasite *Schistosoma mansoni* and bacterium *Helicobacter pylori* produce high levels of IL-10 and Th2-attracting chemokines, leading to a remarkable skewing from Th1 to Th2 immune cell polarization. The underlying mechanism of this switch involves activation of the IKKε- and CYLD-dependent BCL3 signaling pathways. In analogy, recognition of tumor-specific glycosylation patterns by DC-SIGN on DCs might also play a crucial role in the suppression of anti-tumor immune responses against cancer-related fucosylated antigens.

Furthermore, mouse MGL1 (CD301) is a murine CLR exhibiting a similar glycan specificity to DC-SIGN, with specific recognition of the Lewis^a^ and Lewis^x^ glycan structures (126). MGL1 is expressed by murine innate immune cells, such as immature conventional DCs, plasmacytoid DCs, and macrophages. Although MGL1 is considered to be a marker of alternatively activated macrophages (127), its biological role in the recognition of cancer cell-derived Lewis^a^ and Lewis^x^ antigens, as well as in the induction of potential anti-tumor immune responses, needs further elucidation.

Langerin (CD207) is a CLR expressed by human Langerhans cells (LCs). LCs constitute a DC subset in the skin and in the epithelium of mucosal tissues (128), where they act as key modulators in the induction of immune responses against invading pathogens (129). Langerin specifically binds the terminal fucose of Lewis^b^ and Lewis^y^ antigens, while, in contrast to DC-SIGN, it cannot interact efficiently with the internal fucose that is present in the Lewis^a^ and Lewis^x^ trisaccharides (130, 131).

Immature LCs are potent inducers of immune tolerance; however, upon maturation these cells are capable of activating effective antigen-specific immune responses (132). Infiltration of immature CD207^+^/Langerin^+^ DCs in the peritumoral area of invasive cutaneous malignant melanoma (CMM) is correlated with increased tumor growth, a high mitotic rate, and CMM ulcer development, all leading to disease exacerbation and a worse prognosis (133). Also, the infiltration of Langerin^+^ cells in Hodgkin lymphomas and in nasopharyngeal carcinomas has been associated with the already well-characterized, strong immunosuppressive tumor microenvironment in these two types of cancers (134). Nevertheless, more research is required to determine the exact interactions that occur among infiltrating LCs and Lewis^b/y^-expressing tumor cells in the skin or other mucosal tissues.

## Resistance to Chemotherapy

One of the biggest challenges in the treatment of cancer is the development of multidrug resistance to chemotherapy (135). Malignant cells employ a number of different mechanisms in order to survive, evolve, and to become insensitive to anti-cancer drugs. These mechanisms are mainly related to the drug uptake/efflux ratio, activation of certain DNA repair mechanisms, and a successful escape from drug-induced apoptosis (136).

Aberrant glycosylation in cancer, including increased fucosylation, is associated with an EMT in combination with acquiring a multidrug resistance phenotype (137). For example, upregulation of the FUT4, FUT6, and FUT8 enzymes has been implicated in the drug resistance phenotype of the human HCC cell lines BEL7402 and BEL/FU both *in vitro* and *in vivo*. Moreover, it is linked to activation of the PI3K/Akt pathway as well as to the induction of the multidrug-resistance-associated protein 1 (138). In ovarian cancer patients, coexpression of Lewis^y^ and MUC1 is considered as an independent risk factor of chemoresistance and poor prognosis (139).

Compared to the sensitive T47D cells, the drug-resistant breast cancer cell line T47D/ADR overexpresses the FUT4 enzyme. In this case, miR-224-3p acts as a negative regulator of FUT4 gene expression. Importantly, induction of miR-224-3p leads to drug sensitization of T47D/ADR cells *in vitro* and impaired tumor growth of T47D/ADR xenografts *in vivo* (140).

Furthermore, transcriptional upregulation of FUT4, resulting in an increased cell-surface expression of the Lewis^x^ antigen, remains a serious obstacle for the treatment of patients with metastatic colorectal cancer. FUT4/Lewis^x^ overexpression is induced by the RAF-MEK-ERK signaling pathway, and colon cancer cells that are FUT4^+^Lewis^x+^ seem to exhibit significant resistance to the anti-EGFR (cetuximab) and the anti-VEGF (bevacizumab) chemotherapeutical agents (141). Nevertheless, treatment with MEK inhibitors notably suppresses FUT4 expression in primary CRC cells and can be effective for preventing or overcoming primary resistance of patients to cetuximab and bevacizumab.

A better insight into the role of other FUTs or their respective fucosylated epitopes in the acquisition of a drug-resistant phenotype seems to be mandatory. Understanding the exact role of glycosylation-related alterations in the evasion of cancer cells from drug toxicity could lead to the development of novel and more targeted anti-cancer therapies able to fight multiple aspects of the disease simultaneously.

## Lewis Antigens and Cancer Stemness

Cancer stem cells or cancer-initiating cells are defined as a subpopulation of cells within the tumor, possessing the ability to self-renew and to give rise to heterogeneous lineages of cells. Moreover, cancer stem cells are able to regenerate new, continuously growing tumors when injected *in vivo* (142). Due to the aforementioned characteristics and their involvement in multidrug resistance and metastasis (143), cancer stem cells have been placed into high focus of anti-cancer therapy nowadays (144).

The identification of cancer stem cells has proven to be quite a big challenge, especially in the case of solid tumors where these cells are less accessible. In addition, the number of the stem cell isolation assays developed as well as the panel of established, cancer-specific stem cell markers are still quite limited (145). So far, cell surface markers such as CD44, CD133 (prominin-1), Sca-1 (stem cell antigen-1), CD24 (heat-stable antigen), CD29 (integrin β1), CD49f (integrin α6), and ESA (epithelial-specific antigen) have been frequently used for the detection of breast, colorectal, pancreatic, and prostate cancer stem cells (146). Furthermore, high activity of aldehyde dehydrogenases (ALDHs) in stem cells make ALDH another valuable marker to establish cancer stemness (147).

Increased fucosylation has recently been associated with the known cancer stem cell phenotype and has thus been proposed as a potential therapeutic target (148). Elevated levels of fucosylation, driven by the overexpression of pertinent FUTs, GDP-fucose synthetic enzymes, and GDP-fucose transporters, have been identified in pancreatic cancer stem-like (Panc-1-RG CD44^+^/CD24^+^) cells that are resistant to the chemotherapeutic agent gemcitabine (149). Also, upregulation of FUT3 and FUT6 enzymes combined with increased sialyl Lewis^x^ expression has been reported in stem-like (CD44^+^/ALDH^+^) cells derived from oral squamous cell carcinomas. Moreover, this phenotype is correlated with the increased metastatic potential of these cells (150).

Lewis^x^ is highly expressed by gliomas, and a possible role of this epitope as a cancer stem cell selection marker in human glioblastoma (also known as glioblastoma multiforme) has been proposed (151). Lewis^x+^ cells were enriched in human glioma tumor-initiating cell populations in 23/24 of primary glioblastoma specimens examined, fulfilling all the criteria of cancer stem cells. More specifically, Lewis^x+^ cells were highly tumorigenic when injected in the brain of SCID mice and displayed self-renewal and multilineage differentiation properties, giving rise to both Lewis^x+^ and Lewis^x−^ cells, thus, supporting a cellular hierarchy. Interestingly, Lewis^x^ has also been identified as a marker for tumor propagating cells in a mouse model of medulloblastoma (the Patched mutant mouse) (152). However, its potential role as a stem cell marker in other types of human cancers is still unknown.

Significant coexpression of the H2 antigen (CD173) and the Lewis^y^ antigen (CD174) with the cancer stem cell marker CD44 has been identified in breast cancer tissue sections and in breast cancer cell lines (153). Immunoprecipitation experiments revealed that CD44 is the carrier molecule of these two fucosylated antigens, explaining the consistent coexpression observed. Coexpression of H2 and Lewis^y^ with another well-known cancer stem cell marker, CD133, was also reported in this study. These data suggest that the fucosylated H2 and Lewis^y^ epitopes might be used as cancer stem cell enrichment markers in breast carcinomas.

Since fucosylated determinants are overexpressed in almost all types of solid or blood malignancies and their expression is correlated with increased incidence of tissue invasion, metastasis, and multidrug resistance, a potential role of these epitopes in cancer stemness has been suggested. Yet, it is currently still unclear whether Lewis antigens are mere markers of malignant cell transformation or whether they have a direct biological/functional contribution to the cancer stem cell phenotype.

## Future Directions

**Figure 2:**
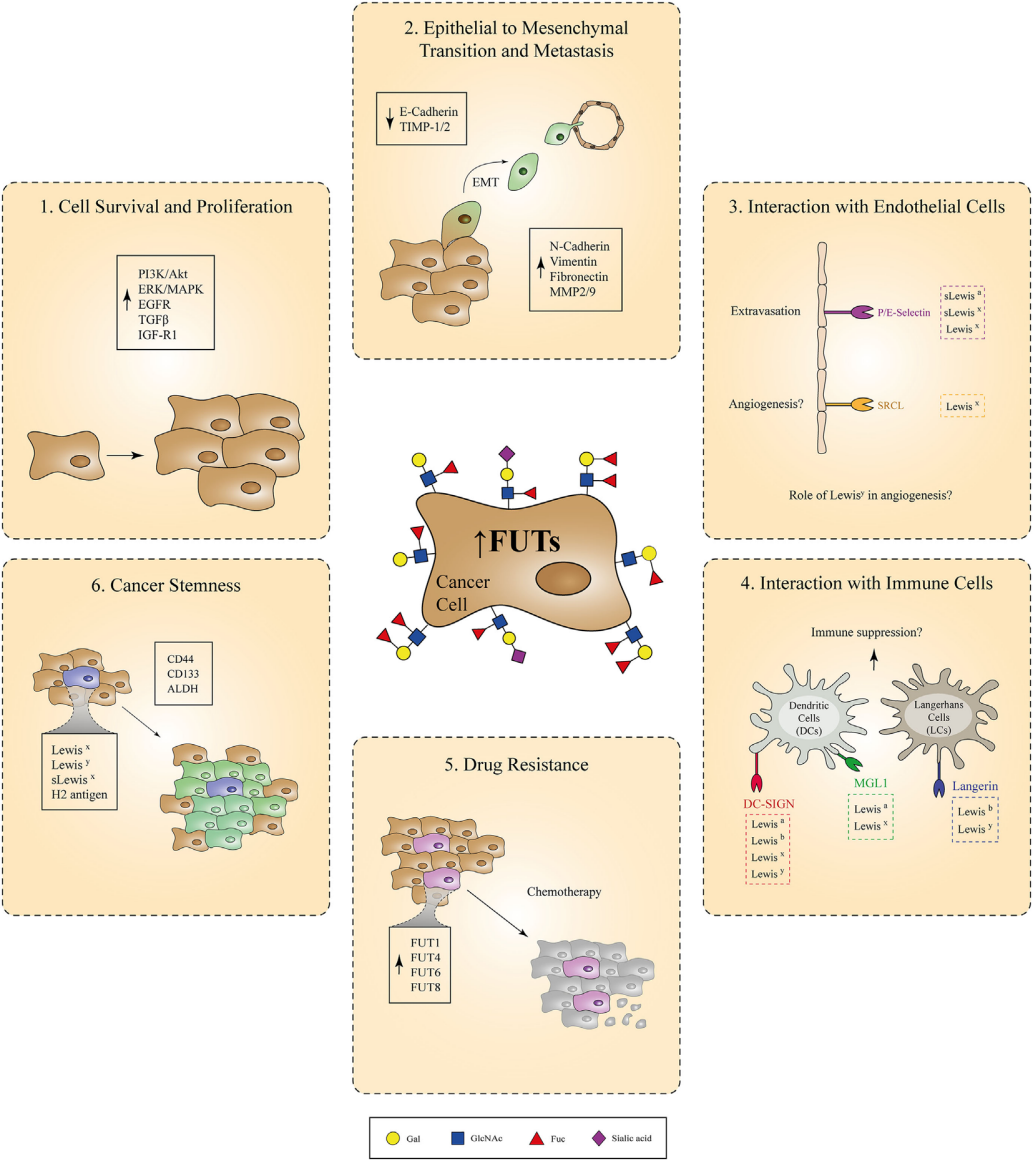
Involvement of fucosylated antigens in different aspects of cancer progression. Due to genetic or epigenetic alterations, overexpression of certain fucosyltransferases (FUTs) by cancer cells leads to increased cell-surface expression of fucosylated Lewis antigens. Aberrant regulation of the fucosylation machinery in cancer cells is causally associated with the acquisition of various tumorigenic properties, such as increased cell survival and proliferation, epithelial to mesenchymal transition, metastasis, resistance to chemotherapy, and cancer stemness. In parallel, interaction of cancer cells overexpressing Lewis antigens with endothelial or immune cells bearing Lewis antigen-specific receptors might also play a critical role during cancer-induced angiogenesis and immunosuppression, respectively.

In summary, Lewis antigens expressed by healthy tissues serve as important adhesion molecules involved in the communication of different cell subtypes, such as epithelial cells, immune cells, and neurons, with their surrounding microenvironment during normal development. Tight regulation of their expression on the cell-surface seems to be necessary, since perturbations in this balance are greatly associated with cancer development and progression. Specifically, overexpression of Lewis epitopes occurs in malignant cells as a direct consequence of genetic or epigenetic alterations, resulting in upregulation of pertinent FUT genes. It is currently well accepted that this overexpression is not just a bystander effect observed during malignant cell transformation. Instead, the increased expression of FUT enzymes and their respective fucosylated determinants enables the acquisition of many functional features of cancer cells related to cell proliferation, EMT/tissue invasion, metastatic potential, interaction with endothelial/immune cells, and resistance to chemotherapy and cancer stemness (Figure 2). These properties are all interconnected and eventually culminate in disease exacerbation. Therefore, high emphasis should be given to the identification of specific anti-cancer therapies that correct the fucosylation machinery within malignant cells and inhibit subsequent tumor growth in patients. Along with the therapeutic applications that target aberrant fucosylation in cancer, some outstanding research questions still remain to be answered:

- *Vascularization*: is there any effect of Lewis antigen overexpression on cancer vascularization *via* binding to CLRs (e.g., SRCL) expressed by endothelial cells? Is there a role of Lewis^y^ in the interaction between endothelial cells and tumor cells?
- *Immune modulation*: do Lewis antigens contribute to the establishment of an immunosuppressive tumor microenvironment through the interaction with CLRs?
- *Gene therapy*: could novel genome engineering approaches (e.g., CRISPR-Cas9) specifically target and reverse the overexpression of FUT genes in cancer cells?

## Author Contributions

AB wrote the manuscript. AB and ER designed the figures. NS, YK, and SV revised and corrected the manuscript.

## Conflict of Interest Statement

The authors declare that the research was conducted in the absence of any commercial or financial relationships that could be construed as a potential conflict of interest.
