# Corrigendum: Fucosylated Antigens in Cancer: An Alliance Toward Tumor Progression, Metastasis, and Resistance to Chemotherapy

**DOI:** 10.3389/fonc.2018.00150

**Published:** 2018-05-11

**Authors:** Athanasios Blanas, Neha M. Sahasrabudhe, Ernesto Rodríguez, Yvette van Kooyk, Sandra J. van Vliet

**Affiliations:** Department of Molecular Cell Biology and Immunology, Cancer Center Amsterdam, VU University Medical Center, Amsterdam, Netherlands

**Keywords:** cancer, glycosylation, fucosylation, fucosyltransferases, Lewis antigens

We recently published our review with the title: “Fucosylated Antigens in Cancer: An Alliance Toward Tumor Progression, Metastasis, and Resistance to Chemotherapy” in Frontiers in Oncology.

However, we noticed that there is a mistake in the abstract of the article. Specifically, the abstract mentions “**endothelial** to mesenchymal transition” instead of “**epithelial** to mesenchymal transition.”

We are submitting this Corrigendum in order to clarify the error and apologize for any confusion by the readers.

The correct sentence should read: “Here, we review the relevance of tumor-overexpressed FUTs and their respective synthesized Lewis determinants in critical aspects associated with cancer progression, such as increased cell survival and proliferation, tissue invasion and metastasis, epithelial to mesenchymal transition, endothelial and immune cell interaction, angiogenesis, multidrug resistance, and cancer stemness.”

The original article has been updated.

## Conflict of Interest Statement

The authors declare that the research was conducted in the absence of any commercial or financial relationships that could be construed as a potential conflict of interest.

